# Predictors of research productivity among physical therapy programs in the United States: an observational study

**DOI:** 10.1186/s12909-020-02133-1

**Published:** 2020-07-11

**Authors:** David M. Rowland, Amanda A. Murphy, Hannah R. Manik, Chris Y. Lane, Deborah L. Givens, Chad E. Cook, Alessandra Narciso Garcia

**Affiliations:** 1 Duke University Division of Physical Therapy, 2200 W Main St, Durham, Durham County, NC 27705 USA; 2 Division of Physical Therapy, Department of Allied Health Sciences, University of North Carolina at Chapel Hill, Chapel Hill, NC 27599 USA; 3 Duke University Division of Physical Therapy, Duke Clinical Research Institute, Durham, Durham County, NC USA; 4 Duke Department of Orthopaedic Surgery, Duke University Division of Physical Therapy, Durham, Durham County, NC USA

**Keywords:** Accreditation, Education, medical, Logistic models, Physical therapists, Research

## Abstract

**Background:**

This study targeted the association of program characteristics of 203 Doctor of Physical Therapy (DPT) programs in the United States (US) reported by the Commission on Accreditation in Physical Therapy Education (CAPTE) in their 2017 Annual Accreditation Report (AAR) with measures of core faculty research volume. The association of institutional, program, and faculty characteristics of an institution with core faculty research volume was investigated.

**Methods:**

This observational study analyzed data provided in the AAR about program research volume. Predictor variables included institutional, program and faculty characteristics. Research volume was measured as a ratio of 1) number of peer-reviewed publications, 2) National Institutes of Health (NIH) funding, and 3) faculty with grants, per number of core faculty. Research volume was stratified by quartiles and analyzed using logistic regression analyses. The highest 25% were analyzed against the lowest 75%.

**Results:**

In the multivariate logistic regression analyses, research Carnegie classification was positively associated with NIH funding (OR = 4.04; 95% CI = 1.92, 8.48) and number of peer reviewed publications (OR = 7.63; 95% CI = 3.39, 17.14). Square footage of research space was positively associated with number of peer reviewed publications (OR = 4.58; 95% CI = 2.08, 10.11). Private status was negatively associated with NIH funding (OR = 0.37; 95% CI = 0.17, 0.83) and faculty holding grants (OR = 0.38; 95% CI = 0.19, 0.76).

**Conclusions:**

There is strong evidence that research culture (e.g., research Carnegie status and dedicated research space) is related to research productivity in DPT programs in the US. Private status was indicative of a non-research intensive environment, which may be reflective of a current trend of small, non-research based private institutions initiating DPT programs.

## Background

An entry-level physical therapy program of education prepares the students to the point of being able to practice as physical therapists in the country of education [1]. Entry requirements, length of education, and the final qualifications obtained vary across countries. In the United States (US), all new graduates are conferred a Doctor of Physical Therapy (DPT) degree. Physical therapists are primary care clinicians in the US with direct access to patient care, and thus, a clinical doctoral-level degree is viewed by the governing body as warranted [2]. The doctoral degree is mandatory in the US to foster lifelong learning, independence, collaboration, and development of the practice. Details of entry to practice requirements may be accessed from the relevant World Confederation for Physical Therapy (WCPT) member organization [1]. Because the data analyzed reflects US DPT programs, the background will be specific to the governing bodies within the US.

The Commission on Accreditation in Physical Therapy Education (CAPTE) [3] collects data annually from all accredited DPT programs in the US and their territories in their Annual Accreditation Report (AAR). They publish a public document with this information, the Aggregate Program Data [4]. CAPTE requires a minimum standard for scholarship for faculty within a DPT program that at least 50% of a program’s core faculty hold an advanced academic doctoral degree beyond a clinically-based DPT [5]. Beyond CAPTE, scholarly activity is a common program requirement for all academic faculty members used to determine the qualitative strength and ranking of a program [6–11]. Scholarly activity refers to much more than traditional research. The APTA has described it as, “creative, communicated, peer-validated intellectual work (scholarship) in any of its several forms (discovery, development, integration, and artistry)” [12].

Scholarly activity is historically low among physical therapy programs [6], although a program’s level of productivity does not seem to influence a program’s National Physical Therapy Examination (NPTE) pass rate in a recent study [7]. To date, the NPTE pass rates are most notably influenced by student-level factors such as mean undergraduate GPA [10]. An older dataset identified a relationship between a non-research Carnegie classification and lower NPTE pass rate [11], but physical therapy programs may have changed significantly in this time and a larger sample is needed to validate the findings. Despite this, programmatic investments have been shown to have a positive effect on NPTE pass rate [13], suggesting that other factors reported in the AAR may differentiate the successes of physical therapy programs beyond NPTE pass rate.

Scholarly activity is a focus of both CAPTE and the American Physical Therapy Association (APTA) in order to provide evidence-based care [5, 14]. Evidence-based care has been thought to reduce unnecessary variability and improve patient care [15]. Evidence that elucidates relationships between program characteristics and magnitude of scholarly activity may be useful to guide DPT programs to align with the APTA’s vision for physical therapy [14]. This study aimed to investigate the association of institutional, program, and faculty characteristics reported in CAPTE’s AAR with standardized research volume by DPT programs within the US.

## Methods

### Study design

This study was an observational design. The REporting of studies Conducted using Observational Routinely-collected Data (RECORD) Checklist was utilized to guide the reporting of this study.

### Data source

The data used in the study, the most recently organized results from the CAPTE AAR, was obtained from CAPTE-accredited DPT programs that graduated students in 2017. The CAPTE AAR provided institutional and general information about the accredited DPT programs, including characteristics pertaining to the institution, program, and faculty. The Institutional Review Board of the University of North Carolina (ID #18–3059) determined that the data does not constitute human subjects research. Thus, informed consent was waived under federal regulations [45 CFR 46.102 (d or f) and 21 CFR 56.102 (c)(e)(l)].

### Institutions information

The institutional data comprised the 203 DPT programs within the US that were accredited at the time of the study. Identifiers were masked for the institutions in the data set to conceal their identities, such that investigators could not link an entry to a specific institution. Selective data about each institution was provided based on the core faculty for the DPT program, defined by the AAR as “those individuals appointed to and employed primarily in the program … with the responsibility and the authority related to the curriculum” [16]. The AAR includes the number of peer reviewed publications, amount of NIH funding (to include the total across all departments receiving money and years), and number of faculty members holding grants, all of which represent research productivity. All data was program-level information, as no individual student data was provided.

### Variables used in the modeling

The CAPTE AAR provided a comprehensive list of variables that had the possibility to be included in this analysis. However, because we were interested in the variables that could be indicators of research productivity, only those reflective of institutional, program, and faculty characteristics were considered. An additional glossary describes these terms in more detail (see Additional file 1 [2, 17]).

### Independent variables

Variables were chosen that were a) reflective of the institutions, b) reflective of the institutions’ program format, and c) reflective of faculty characteristics. These variables were separated into institutional, program and faculty characteristics for interpretation of the analyses.

### Institutional characteristics

The institutional characteristics included four variables: Carnegie classification (research universities/doctorate and research, all others [other health professional schools, medical schools, and medical centers and masters colleges and universities]), private status, traditional institution type, and student body size (whole institution). All variables were reported as categorical data, dichotomized as follows: a) Carnegie classification (research universities/doctorate and research versus all others), b) private status (private versus public), c) traditional institution status (traditional [Academic Health Science Center, Liberal Arts College (4-year), and Liberal Arts University] versus all others [Proprietary, Osteopathic Medical School, Professional and Technological University]) and d) student body size (> 10,000 versus < 10,000).

### Program characteristics

The program characteristics included nine variables: > 6 year program format, number of terms, total program length, number of credits, classroom education hours, hybrid curriculum, operating budget, total number of courses, and square footage of research space. Program format, operating budget and type of curriculum were reported as categorical data, dichotomized as follows: a) > 6 year program format (> 6 years, 6 years), b) operating budget (interquartile range upper 25%, interquartile range lower 75%) and c) curriculum model (traditional versus all others [hybrid, systems-based, problem-based, modified problem-based, guide-based]). Number of terms, total program length, number of credits, classroom education hours, total number of courses and square footage of research space were reported as continuous data by mean and standard deviation (SD).

### Faculty characteristics

The faculty characteristics included four variables: total number of vacancies, faculty turnover, faculty to student ratio, and total full-time equivalents. All four variables were reported as continuous data by mean and SD.

### Dependent variables (outcome variables of interest)

In order to describe the scholarly culture of the institutions in the dataset from CAPTE, the outcomes representing a proxy for productivity were considered, including peer reviewed publications, National Institutes of Health (NIH) funding, and total number of faculty within the DPT program who reported they had grants were each standardized per core faculty member. Stratification was performed for these three outcomes to separate the top 25% from the bottom 75%, with the intent to capture the highest performing research-intensive institutions in the upper quartile [18]. To our knowledge, no single value in the literature provides a meaningful cutoff to represent research productivity. Indeed, only a few physical therapy programs report high productivity, and within the AAR a median score of ‘zero’ is present for publications and NIH funding. Consequently, instead of splitting the variables at the median [19, 20], we elected the same method (quartile rank/25–75%) used in previous medical research [21–23]. These three outcomes were used to associate research productivity to variables in the dataset that might modify the outcomes.

### Statistical analysis methods

All of the analysis used in this study was performed in using Statistical Package for the Social Sciences (SPSS) version 25.0 (IBM Corp. Armonk, NY, USA). Descriptive statistics (mean/SD and frequencies) were used to summarize the institutional, program and faculty characteristics (both for dependent and independent variables). To assess multicollinearity in the modelling a correlation analyses were performed between the 17 independent variables. Correlation was defined as a negligible positive or negative correlation (*r* = 0.00 to 0.30), low positive or negative correlation (*r* ≥ 0.30 to 0.50), moderate positive or negative correlation (*r* ≥ 0.50 to 0.70), high positive or negative correlation (> 0.70 to 0.90) and very high positive or negative correlation (> 0.90 to 1.0) [24, 25]. We decided to remove variables with correlations ≥0.70 to not influence models. A *p*-value < 0.05 was considered significant.

A comparative analysis was performed to relate the independent variables with the outcomes reflective of research productivity. Univariate logistic regression analyses were performed between the three outcome measures and each of the 17 independent variables. For each univariate analysis of the independent variables, *p*-values, odds ratios (OR), 95% confidence intervals (CI) and the percentage of explained variance (Nagelkerke’s R^2^ values) were reported. An OR measures the association between an outcome and exposure to a particular variable, with values > 1.0 indicating that exposure is associated with higher odds of the outcome [26]. Nagelkerke’s R^2^ values are similar to an R-squared value and indicate the power of the model [27]. Associations in univariate analyses with significant *p*-values were considered in a multivariate backward stepwise logistic regression analyses for each of the three outcome measures of research productivity. In each of these three multivariate models, a *p*-value < 0.05 was considered significant. We adopted the criterion proposed to Harrell et al., equivalent to a minimum of 10 to 20 events per variable for logistic regression [28, 29].

## Results

### Institutional, program and faculty characteristics

Institutional, program and faculty characteristics of 203 DPT programs are shown in Table 1. Although there were significant associations among many independent variables, none of the associations were near the predefined threshold of 0.7 (please see Additional file 2 at the end of the document). For these reasons, we did not remove or combine any of the independent variables.

**Table 1:** Descriptive institutional, program and faculty characteristics (*n* = 203)

Variables	Whole sample (*n* = 203)	Number of peer reviewed publications per core faculty member		Faculty with grants per core faculty member (SD)		NIH funding per core faculty member	
		Top 25%	Bottom 75%	Top 25%	Bottom 75%	Top 25%	Bottom 75%
Institution characteristics							
Carnegie Classification (research status)	83 (40.9)	39 (19.2)	43 (21.1)	29 (14.2)	54 (26.6)	41 (20.2)	42 (20.6)
Private Status	99 (48.8)	16 (7.9)	81 (39.9)	15 (7.4)	83 (40.9)	20 (9.9)	78 (38.5)
Traditional Institution Status	187 (92.1)	48 (23.6)	136 (67.0)	46 (22.7)	140 (69.0)	55 (27.1)	131 (64.5)
Student Body Size (> 10.000)	73 (36.0)	34 (28.0)	37 (30.4)	27 (22.2)	46 (37.8)	36 (29.6)	37 (30.4)
Program Characteristics							
> 6 Year Program Format	179 (88.2)	46 (22.7)	130 (64.1)	48 (23.7)	130 (64.1)	54 (26.6)	124 (61.1)
Number of Terms (SD)	8.6 (1.2)	8.6 (1.2)	8.6 (1.4)	8.6 (1.3)	8.6 (1.2)	8.6 (1.1)	8.6 (1.3)
Total Program Length (SD)	122.7 (12.0)	124.3 (11.6)	122.5 (11.8)	122.4 (12.5)	122.8 (11.9)	124.4 (12.9)	122.0 (11.6)
Number of Credits (SD)	112.6 (29.0)	113.3 (34.2)	112.3 (27.4)	111.1 (37.3)	113.1 (25.8)	112.0 (34.6)	112.9 (26.5)
Classroom Education Hours (SD)	1804.1 (422.9)	1734.9 (403.8)	1827.6 (431.8)	1853.6 (438.7)	1787.7 (419.0)	1791.7 (452.4)	1809.6 (413.2)
Hybrid Curriculum	150 (74.0)	33 (16.3)	115 (56.7)	36 (17.8)	114 (56.2)	45 (22.2)	105 (51.8)
Operating Budget (upper 25%)	51 (86.4)	16 (27.1)	34 (57.6)	17 (28.8)	34 (57.6)	26 (44.1)	25 (42.4)
Total Number of Courses (SD)	40.3 (7.4)	39.7 (7.2)	40.5 (7.5)	40.4 (6.5)	40.3 (7.7)	40.0 (7.7)	40.4 (7.3)
Square Footage of Research Space (SD)	3165.8 (3152.4)	5247.1 (4225.1)	2475.2 (2297.5)	3881.1 (3256.8)	2940.4 (3095.5)	4491.3 (3250.6)	2636.0 (2960.8)
Faculty Characteristics							
Total Number of Vacancies (SD)	0.6 (0.7)	0.7 (0.1)	0.6 (0.1)	0.5 (0.8)	0.6 (0.7)	0.7 (0.8)	0.5 (0.7)
Faculty Turnover (SD)	5.6 (7.6)	4.5 (0.9)	5.9 (8.0)	7.0 (8.5)	5.0 (7.3)	5.5 (7.5)	5.6 (7.7)
Faculty to Student Ratio (SD)	11.4 (3.3)	10.6 (3.1)	11.6 (3.3)	11.6 (2.6)	11.3 (3.5)	10.7 (3.7)	11.6 (3.1)
Total Full Time Equivalents (SD)	11.1 (4.5)	12.4 (5.6)	10.8 (3.9)	11.0 (3.9)	11.2 (4.6)	13.9 (5.5)	10.0 (3.2)

Variables represent number (%) unless otherwise noted

### Research productivity - number of peer reviewed publications per core faculty member

Four of the 17 independent variables displayed significance (*p*-values < 0.05) when associated with the number of peer reviewed publications per core faculty member in the univariate logistic regression analysis (Table 2).

**Table 2:** Univariate logistic regression analyses for peer reviewed publications, NIH funding and faculty with grants

	Outcomes								
	Number of Peer Reviewed Publications per Core Faculty Member			NIH funding per core faculty member			Faculty with grants per core faculty member		
Variables	OR (95% CI)	*P* value	R^2^	OR (95% CI)	*P* value	R^2^	OR (95% CI)	*P* value	R^2^
Institution Characteristics									
Carnegie Classification	9.80 (4.49, 21.36)	**< 0.01**	0.27	5.48 (2.83, 10.61)	**< 0.01**	0.18	2.37 (1.24, 4.52)	**< 0.01**	0.05
Private Status	0.42 (0.21, 0.83)	**0.01**	0.05	0.43 (0.23, 0.80)	**< 0.01**	0.05	0.34 (0.17, 0.68)	**< 0.01**	0.07
Traditional Institution Type	5.29 (0.68, 41.16)	0.11	0.03	1.26 (0.39, 4.08)	0.70	0.00	0.72 (0.24, 2.19)	0.57	0.00
Student Body Size	1.00 (0.98, 1.01)	0.69	0.00	1.00 (0.98, 1.01)	0.66	0.00	1.00 (0.97, 1.02)	0.71	0.00
Program Characteristics									
> 6 Year Program Format	2.48 (0.71, 8.69)	0.16	0.02	1.66 (0.59, 4.66)	0.34	0.01	2.59 (0.74, 9.06)	0.14	0.02
Number of Terms	1.02 (0.79, 1.32)	0.88	0.00	1.04 (0.81, 1.32)	0.77	0.00	1.04 (0.80, 1.34)	0.78	0.00
Total Program Length	1.01 (0.99, 1.04)	0.36	0.01	1.02 (0.99, 1.04)	0.20	0.01	1.00 (0.97, 1.02)	0.81	0.00
Number of Credits	1.00 (0.99, 1.01)	0.84	0.00	1.00 (0.99, 1.01)	0.84	0.00	1.00 (0.99, 1.01)	0.66	0.00
Classroom Education Hours	1.00 (1.00, 1.00)	0.19	0.01	1.00 (1.00, 1.00)	0.79	0.00	1.00 (1.00, 1.00)	0.34	0.01
Hybrid Curriculum	0.65 (0.32, 1.31)	0.22	0.01	1.16 (0.58, 2.36)	0.67	0.00	0.78 (0.38, 1.58)	0.49	0.00
Operating Budget	1.18 (0.21, 6.73)	0.86	0.00	3.12 (0.58, 16.94)	0.19	0.04	1.50 (0.27, 8.24)	0.64	0.01
Total Number of Courses	0.99 (0.94, 1.03)	0.52	0.00	0.99 (0.95, 1.03)	0.71	0.00	1.00 (0.96, 1.05)	0.96	0.00
Square Footage of Research Space	6.63 (3.23, 13.63)	**< 0.01**	0.19	3.24 (1.65, 6.36)	**< 0.01**	0.08	2.40 (1.20, 4.80)	**0.01**	0.04
Faculty Characteristics									
Total Number of Vacancies	1.24 (0.82, 1.88)	0.32	0.01	1.46 (0.98, 2.16)	0.06	0.02	0.89 (0.58, 1.38)	0.61	0.00
Faculty Turnover	0.97 (0.93, 1.02)	0.26	0.01	1.00 (0.96, 1.04)	0.93	0.00	1.03 (0.99, 1.07)	0.11	0.02
Faculty to Student Ratio	0.91 (0.82, 1.00)	0.06	0.03	0.92 (0.84, 1.01)	0.09	0.02	1.03 (0.93, 1.14)	0.54	0.00
Total Full Time Equivalents	1.08 (1.01, 1.16)	**0.03**	0.04	1.24 (1.14, 1.35)	**< 0.01**	0.21	0.99 (0.92, 1.07)	0.80	0.00

Bolded *p*-values represent significant findings

These four variables were entered into the backward multivariate model. Only Carnegie classification and square footage of research space remained predictors for this outcome. The presence of high number of peer reviewed publications per core faculty member was associated with a research Carnegie classification (OR = 7.63, 95% CI 3.39, 17.14, *p* = < 0.01) and square footage of research space (OR = 4.58, 95% CI 2.08, 10.11, *p* = < 0.01) (Table 3).

**Table 3:** Multivariate logistic regression modeling including predictor variables

Outcomes	Predictor variables	OR (95% CI)	Nagelkerke (*R*^*2*^)	Individual *p* value
Number of Peer Reviewed Publications per Core Faculty Member	Carnegie Classification	7.63 (3.39, 17.14)	0.36	**< 0.01**
	Square Footage of Research Space	4.58 (2.08, 10.11)		**< 0.01**
NIH funding per core faculty member	Carnegie Classification	4.04 (1.92, 8.48)	0.37	**< 0.01**
	Private Status	0.37 (0.17, 0.83)		**0.02**
Faculty with grants per core faculty member	Private Status	0.38 (0.19, 0.76)	0.10	**< 0.01**

Bolded *p*-values represent significant findings

### Research productivity - NIH funding per core faculty member

Four of the 17 independent variables displayed significance against NIH funding per core faculty member in the univariate logistic regression analysis (Table 2). The presence of high NIH funding per core faculty member was associated with a research Carnegie classification (OR = 4.04, 95% CI 1.92, 8.48, *p* = < 0.01) (Table 3). The presence of low NIH funding per core faculty member was associated with private status (OR = 0.37, 95% CI 0.17, 0.83, *p* = 0.02) (Table 3).

### Research productivity - faculty with grants per core faculty member

Three of the 17 independent variables displayed significance against faculty with grants per core faculty member in the univariate logistic regression analysis (Table 2). The presence of low faculty with grants per core faculty member was associated with private status (OR = 0.38, 95% CI 0.19, 0.76, *p* = < 0.01) (Table 3).

## Discussion

### Summary of findings

This study investigated the association of institutional, program, and faculty characteristics reported in CAPTE’s AAR with standardized research volume by DPT programs within the US. The data in this study demonstrates the higher affinity for scholarly activity in physical therapy within research intensive parent institutions. This is evidenced by the positive relationships of research Carnegie classification and square footage of research space with scholarly activity. In addition, greater full-time equivalents in a program has a positive relationship with scholarly activity in the univariate regression but this did not remain significant in the multivariate regression. This suggests that, when accounting for confounding variables such as employing a greater number of faculty to support a larger PT program, full-time equivalents are not inherently research specific.

### Research culture

CAPTE places minimal standards on scholarly activity present in physical therapy programs; accredited programs are required to employ at least 50% of faculty holding an advanced academic doctoral degree [5]. An institutional culture of research and investment in the students and faculty reflects in increased research productivity [30]. Growth in private, non-research intensive institutions does not appear to add to such productivity [9]. Using the best available evidence is a core pillar of utilizing evidence-based practice in physical therapy, as noted by the APTA [15]. That is, research advances the field of physical therapy, developing more effective patient management strategies for future clinicians. Building a culture of research within the physical therapy profession can begin in physical therapy schooling. It is worth noting, however, that research productivity does not completely ensure students adopt evidence-based practice. As well, it is unclear by the data provided in the AAR whether the scholarly activity conducted by physical therapy program faculty involves or does not involve the practice. Further research is needed to clarify this relationship. The NIH has previously warned of the extinction of the clinical scientist [31], citing the recent increase in clinical training requirements, duration and associated cost. Physical therapy, a clinical practice, must overcome this challenge of engaging more physical therapists in clinical research to drive the practice forward [14, 15]. Certain characteristics have been defined among academic institutions outside of physical therapy that result in an increase of scholarly productivity, most notably collaboration between faculty, time to dedicate to research and leadership in a research environment [32]. Creating an environment that fosters research at the institution level and connecting the students to this environment has been proposed to build student interest in research [33]. As such, creating an environment that fosters research at the DPT programmatic level could then build a future of DPT clinical scientists.

### Private universities

The number of private physical therapy programs has grown from 109 in 2013 to 140 in 2018 (28.44%), despite the more muted growth from 110 to 120 public physical therapy programs (9.09%) over that time [4]. Given the relationship found in this study, there is concern that the steeper growth in private institutions is not significantly adding to the evidence in physical therapy practice. Riley et al. noted that the majority of these new private programs are not housed within research intensive environments [9]. As per CAPTE’s position on scholarship, faculty should serve as role models for students and bridge the connection between research and practice [34]. We find the proliferation of non-research based private institutions intriguing and feel this deserves further exploration.

### Study limitations

This study was completed with limitations in the dataset. There are certainly other factors which affect scholarly activity and could not be captured as a variable in this study. As well, creating categorical variables from continuous data always leads to a loss of sensitivity in the data due to loss of information analyzed. It also risks leaving confounding variables present. It is difficult to quantify the impact of scholarly activity within the programs, and this is a prohibitive limitation of the AAR variables. Whether or not this increased research productivity results in encouragement for the student body to pursue conducting research in physical therapy remains to be seen. As well, there is no clarification as to the type of research generated, whether or not this is specific to the field of physical therapy, and the clinical application of such research.

### Suggestions for future studies

With the ongoing increase in programs accredited by CAPTE, future studies should examine the changes in scholarly activity by program characteristics over time. It also remains to be investigated as to how the scholarly activity of a physical therapy program affects the students’ future affinity for research.

## Conclusions

This study found that certain characteristics exist within physical therapy programs which build a research-intensive environment. These characteristics suggest that physical therapy research, which is suggested to advance the profession forward [14, 15], is conducted at a higher level within public institutions built around research collection. This raises the point that research-intensive institutions further the profession by creating a culture of research among the faculty of the physical therapy program.

## Supplementary information

**Additional file 1.** Description of dependent and independent variables.

**Additional file 2.** Multicollinearity results between independent variables [reported by Pearson correlation (r)].

## Data Availability

The data that support the findings of this study are available from CAPTE but restrictions apply to the availability of these data, which were used under license for the current study, and so are not publicly available. Data are however available from the authors upon reasonable request and with permission of CAPTE.

## Abbreviations

US: United States
DPT: Doctor of Physical Therapy
WCPT: World Confederation for Physical Therapy
CAPTE: Commission on Accreditation in Physical Therapy Education
AAR: Annual Accreditation Report
NIH: National Institutes of Health
NPTE: National Physical Therapy Examination
RECORD: REporting of studies using Observational Routinely-collected Data
SPSS: Statistical Package for the Social Sciences
SD: Standard deviation
APTA: American Physical Therapy Association
