# Supplementary material for: Predictors of research productivity among physical therapy programs in the United States: an observational study
Source: BMC Med Educ. 2020 Jul 11;20:216. doi: 10.1186/s12909-020-02133-1 (PMC7353740; doi:10.1186/s12909-020-02133-1)
Supplement: Supplementary file 1 — Additional file 1. Description of dependent and independent variables. [file 12909_2020_2133_MOESM1_ESM.docx]

Additional file 1. Description of dependent and independent variables.

**Dependent variables (outcome measures)**

1. Peer reviewed publications per core faculty member - refers to the number of peer reviewed publications generated by core faculty of the PT program.
2. National Institute of Health (NIH) funding per core faculty member - refers to the total amount of funding received by the NIH for core faculty of the PT program.
3. Faculty with grants per core faculty member - refers to the number of research grants held by core faculty members.

**Independent variables**

- Carnegie classification - a measure designed to classify institutions of higher education based on their highest degree awarded and capacity to award degrees; the classifications of PT programs are research universities, doctorate and research universities, other health professional schools, medical schools, and medical and masters colleges and universities.
- Private status - the funding status of each PT program, whether it is a public institution which receives funding from the state government of which the institution resides, or a private institution which does not receive such funding.
- Traditional institution status - the teaching status of each PT program, whether it is a traditional teaching model (Academic Health Science Center, Liberal Arts College (4-year), and Liberal Arts University) or an alternative model (Proprietary, Osteopathic Medical School, Professional and Technological University).
- Student body size - the number of students of the entire institution in which the PT program resides; this was reported as either >10,000 students of the entire institution or <10,000 students of the entire institution; sadly, this does not capture student body size of the PT program itself, but is a marker for total institution size.
- >6 year program format - the standard model in the US is a 4+3 year model, in which students gain an undergraduate 4-year degree and move onto the 3-year doctoral degree either within the same or a different institution; an alternative model exists of 3+3 year model, in which students complete 3 years of undergraduate studies and advance to 3 years of graduate studies within the same institution, and are accepted to both upon starting the initial undergraduate teachings.
- Total number of terms - this is the total number of terms, either semesters, trimesters, or quarters, completed in the institution, which is related to but is not entirely indicative of program length.
- Total program length - this is the number of weeks of education completed in the PT program from start to finish, which is a marker of program length.
- Number of credits - this is the number of credits completed in the doctoral degree, which can vary based on elective educational courses and length of program; this is not a standard measure and is based on the courses that universities utilize in the doctorate.
- Classroom education hours - this is the total number of hours spent in the classroom during the didactic portion of the program, which varies widely by program.
- Curriculum model - refers to the style of curriculum, either traditional classroom based or other (hybrid, systems-based, problem-based, modified problem-based, and guide-based).
- Operating budget (upper 25%) - this includes the programs that fit into the top 25% of programs by operating budget; the operating budget was dichotomized into a top 25%/bottom 75% due to a lack of normality in the data and a marked difference in the top 25% of program operating budgets.
- Total number of courses - this is the number of courses, both mandatory and elective, built into the PT program available to students; this is not a standard measure and varies per program.
- Square footage of research space - this is the total amount of research space available to students and/or faculty for conduction of research.
- Total number of vacancies - total number of core faculty vacancies reported at the time of AAR data collection within the PT program.
- Faculty turnover - this is the total number of core faculty that left the program during the year of AAR data collection within the PT program.
- Faculty to student ratio - this is the ratio of core faculty to student ratio within the PT program.
- Total full-time equivalents - the total number of faculty working at a full-time status, which includes fractional additions in the case of part-time core faculty (i.e. a part-time faculty member working at 50% of what the institution defines at full-time would count as 0.5 FTE).

**Other key terms**

- Core faculty member - typically a full-time appointed employee, but may be part-time under certain circumstances, a core faculty member is a faculty that is employed primarily by the institution with direct control over the design and implementation of the curriculum; this includes the program director, the director of clinical education, and other faculty who report directly to the program director.
