# Supplementary material for: Predictors of research productivity among physical therapy programs in the United States: an observational study
Source: BMC Med Educ. 2020 Jul 11;20:216. doi: 10.1186/s12909-020-02133-1 (PMC7353740; doi:10.1186/s12909-020-02133-1)
Supplement: Supplementary file 2 — Additional file 2. Multicollinearity results between independent variables [reported by Pearson correlation (r)]. [file 12909_2020_2133_MOESM2_ESM.docx]

Additional file 2. Multicollinearity results between independent variables [reported by Pearson correlation *(r)*].

| Variables | 1 | 2 | 3 | 4 | 5 | 6 | 7 | 8 | 9 | 10 | 11 | 12 | 13 | 14 | 15 | 16 | 17 |
| --- | --- | --- | --- | --- | --- | --- | --- | --- | --- | --- | --- | --- | --- | --- | --- | --- | --- |
| 1. Carnegie classification | ------ | -0.31** | 0.20** | -0.05 | 0.05 | 0.03 | -0.00 | -0.03 | -0.04 | -0.00 | -0.16 | 0.04 | 0.21** | 0.10 | -0.08 | -0.14* | 0.17* |
| 2. Private/public status | -0.31** | ------ | -0.30** | 0.06 | 0.31** | -0.05 | -0.08 | -0.05 | -0.01 | -0.00 | 0.26** | -0.07 | -0.16* | -0.07 | -0.11 | 0.15* | 0.10 |
| 3. Traditional institution type | 0.20** | -0.30** | ------ | 0.02 | 0.00 | -0.10 | 0.04 | -0.02 | -0.04 | -0.00 | -0.09 | -0.05 | 0.08 | -0.01 | -0.06 | -0.04 | -0.05 |
| 4. Student body size | -0.05 | 0.06 | 0.02 | ------ | 0.02 | 0.08 | -0.01 | 0.01 | 0.01 | -0.11 | -0.27** | -0.01 | -0.06 | -0.05 | -0.05 | 0.01 | -0.04 |
| 5. Program format | 0.05 | 0.31** | 0.00 | 0.02 | ------ | -0.03 | -0.03 | 0.24** | -0.01 | -0.04 | -0.10 | -0.03 | 0.07 | 0.00 | 0.08 | -0.17* | -0.00 |
| 6. Number of terms | 0.03 | -0.05 | -0.10 | 0.08 | -0.03 | ------ | 0.33** | 0.15* | 0.04 | 0.08 | 0.16 | 0.17* | 0.00 | 0.00 | 0.06 | 0.01 | 0.04 |
| 7. Total program length | -0.00 | -0.08 | 0.04 | -0.01 | -0.03 | 0.33** | ------ | -0.00 | 0.10 | 0.04 | 0.17 | 0.14* | -0.07 | -0.05 | 0.04 | 0.07 | 0.04 |
| 8. Number of credits | -0.03 | -0.05 | -0.02 | 0.01 | 0.24** | 0.15* | -0.00 | ------ | 0.12 | -0.00 | -0.08 | 0.12 | 0.01 | -0.21** | -0.03 | -0.12 | -0.01 |
| 9. Classroom education hours | -0.04 | -0.01 | -0.04 | 0.01 | -0.01 | 0.04 | 0.10 | 0.12 | ------ | -0.10 | -0.26* | 0.21** | -0.07 | -0.17* | 0.04 | 0.04 | 0.05 |
| 10. Hybrid type of curriculum | -0.00 | -0.00 | -0.00 | -0.11 | -0.04 | 0.08 | 0.04 | -0.00 | -0.10 | ------ | -0.10 | 0.04 | 0.01 | 0.06 | 0.02 | 0.03 | 0.12 |
| 11. Operating budget (25^th^) | -0.16 | 0.26** | -0.09 | -0.27** | -0.10 | 0.16 | 0.17 | -0.08 | -0.26* | -0.10 | ------ | 0.19 | 0.13 | -0.07 | -0.14 | 0.07 | 0.32* |
| 12. Total number of courses | 0.04 | -0.07 | -0.05 | -0.01 | 0.07 | 0.17* | 0.14* | 0.12 | 0.21** | 0.04 | 0.19 | ------ | -0.00 | -0.03 | 0.03 | 0.23 | 0.07 |
| 13. Square footage research space | 0.21** | -0.16* | 0.08 | -0.06 | 0.07 | 0.00 | -0.07 | 0.01 | -0.07 | 0.01 | 0.13 | -0.00 | ------ | 0.14* | -0.02 | -0.07 | 0.31** |
| 14. Total number of vacancies | 0.10 | -0.07 | -0.01 | -0.05 | 0.00 | 0.00 | -0.05 | 0.21** | -0.17* | 0.06 | -0.07 | -0.03 | 0.14* | ------ | 0.32** | 0.02 | 0.14* |
| 15. Faculty turnover | -0.08 | -0.11 | -0.06 | -0.05 | 0.08 | 0.06 | 0.04 | -0.03 | 0.04 | 0.02 | -0.14 | 0.03 | -0.02 | 0.32** | ------ | 0.10 | -0.02 |
| 16. Faculty to student ratio | -0.14* | 0.15* | -0.04 | 0.01 | -0.17* | 0.01 | 0.07 | -0.12 | 0.04 | 0.03 | 0.07 | 0.23 | -0.07 | 0.02 | 0.10 | ------ | -0.19** |
| 17. Total full time equivalents | 0.17* | 0.10 | -0.05 | -0.04 | -0.00 | 0.04 | 0.04 | -0.01 | 0.05 | 0.12 | 0.32* | 0.07 | 0.31** | 0.14* | -0.02 | -0.19** | ------ |

*Correlation is significant at the 0.05 level (2-tailed).

**Correlation is significant at the 0.01 level (2-tailed).
